# Physiological and Genetic Aspects of Resistance to Abiotic Stresses in *Capsicum* Species

**DOI:** 10.3390/plants13213013

**Published:** 2024-10-28

**Authors:** Xiaolin Zhang, Xiuming Ma, Shihui Wang, Shumei Liu, Shaochuan Shi

**Affiliations:** Shandong Key Laboratory of Bulk Open-field Vegetable Breeding/Ministry of Agriculture and Rural Affairs Key Laboratory of Huang Huai Protected Horticulture Engineering, Institute of Vegetables, Shandong Academy of Agricultural Sciences, Jinan 250100, China; 15895977531@163.com (X.Z.); 15275381908@163.com (X.M.); zwsh1009@163.com (S.W.); lsmei78@126.com (S.L.)

**Keywords:** pepper, abiotic stress, temperature stress, saline–alkali stress, water stress, heavy metal stress, molecular breeding, biotic stress

## Abstract

Abiotic stress is one of the key factors harming global agriculture today, seriously affecting the growth and yield of vegetables. Pepper is the most widely grown vegetable in the world, with both high nutritional and economic values. Currently, the increase in global extreme weather events has heightened the frequency of abiotic stresses, such as drought, high and low temperatures, waterlogging, and high salt levels, which impairs pepper growth and development, leading to its reduced yield and quality. In this review, we summarize the research progress on the responses of pepper to abiotic stress in recent years in terms of physiology, biochemistry, molecular level, and mitigation measures. We then explore the existing problems and propose future research directions. This work provides a reference for the cultivation and development of new pepper varieties resistant to abiotic stress.

## 1. Introduction

*Capsicum* species are native to the tropical regions of Central and South America and can be either an annual or perennial plant. *Capsicum* is favored by consumers for its rich nutritional value and flavor. Peppers contain abundant carotenoids, vitamin C, vitamin E, capsaicin, flavonoids, glucose, malic acid, and other substances beneficial to human health [1]. Peppers are widely used, not only for fresh consumption, drying, brewing, and ornamental purposes but also for extracting capsaicin and capsanthin, which are essential seasonings and additives in modern food production. Capsaicin is also used as a pain reliever in various chemotherapy and radiation treatments, and research has suggested its potential in reducing the risk of cancer [2]. As an important cash crop, peppers are cultivated worldwide [3].

Current research on pepper mainly focuses on the control of disease and pests, horticultural traits, flavor compounds, and pharmacology of the extract. However, peppers are often exposed to adverse abiotic factors, such as extreme temperatures, salinity, drought, flooding, and heavy metal contamination, which cause reduced yield and degraded quality [4]. These abiotic stresses often occur simultaneously or sequentially and are detrimental to pepper growth and reproduction. The signaling responses to abiotic stress conditions are complex, combining molecular, cellular, and physiological mechanisms in plants [5]. In this paper, the physiology, biochemistry, molecular mechanisms, and related genetic responses of pepper to temperature, saline–alkali, water, and heavy metal stresses are reviewed. The corresponding mitigation measures are integrated, and breeding directions are proposed to provide a theoretical reference for the development and utilization of pepper resources and the cultivation of resistant varieties.

## 2. Temperature Stress

Pepper is sensitive to temperature, and the optimal growth temperature is 25–30 °C. Changes in high and low temperatures can affect physiological growth (particularly at the reproductive stage), fruit germination, firmness, fruit maturity, metabolite accumulation rate, and capsaicin synthesis [6].

### 2.1. Physiological Responses to Temperature Stress in Capsicum

During the germination stage, heat stress can reduce germination and emergence rates and the root and shoot growth of germinated seedlings of pepper, resulting in seedling deformity and decreased seedling vigor [7]. The extension of high-temperature treatment time has a negative impact on plant height, stem diameter, leaf number, root length, and the dry and fresh weight of pepper seedlings, with the negative effect on heat-sensitive varieties significantly heavier than that of heat-tolerant varieties [8]. High temperatures during anthesis can reduce the pollen activity of pepper, lead to flower abortion or sterile flowers [9], and affect fruit morphology [10]. During the reproductive stage, high-temperature stress affects the proportion of flowers that form fruit (fruit setting rate), and compared with normal temperatures (33/21 °C day/night), high temperatures (38/30 °C day/night) can significantly reduce the fruit weight (51.6%), fruit diameter (25%), fruit length (30%), and the number of seeds per fruit (57%) of bell pepper plants [11]. High temperature affects pepper fruit development, coloring, nutritional quality, and yield, and the content of capsaicin and ascorbic acid (vitamin C) is significantly reduced [12]. Under low-temperature stress, pepper exhibits decreased nutrient composition, metabolic imbalance, impaired organ function in females, and reduced pollen amount per flower. This consequently affects growth, development, and yield [13]. Severe stress can even lead to the death of pepper plants and cause serious economic losses. When the ambient temperature is below 10 °C, especially during storage or transportation, peppers are susceptible to low-temperature damage, resulting in developmental retardation, sepia-colored pepper calyx, and dented peel [14]. The physiological and biochemical effects of temperature stress on pepper are mainly manifested in osmoregulatory substances, the protective enzyme system, membrane lipid peroxidation, and photosynthesis. Under high or low-temperature stress, photosystem II activity is reported to decrease in pepper [15,16].

The phenotype and physiology of pepper seedlings change under high-temperature stress. After high-temperature treatment, the activities of osmotic regulation substances (proline (Pro) and soluble sugar), antioxidant enzymes (superoxide dismutase (SOD) and peroxidase (POD)), catalase (CAT), ascorbate peroxidase (APX), dehydroascorbate reductase (DHAR), and glutathione transferase (GST) in the leaves of heat-tolerant varieties are reported to be significantly higher than those of heat-sensitive varieties, while the content of malondialdehyde (MDA) is significantly lower [17]. The response of pepper seedlings to high temperatures varies with the temperature range and duration. As the high-temperature duration is extended, the stomatal conductance (Gs), intercellular CO_2_ concentration (Ci), and transpiration rate (Tr) of heat-tolerant varieties are significantly higher than those of heat-sensitive varieties, and the content of proline in heat-tolerant varieties is higher than that in heat-sensitive varieties [18]. Therefore, under high-temperature stress conditions, heat-tolerant varieties can achieve a stable photosynthetic rate by increasing Tr and Pro contents and recover rapidly from the damage caused by high temperature. The accumulation of antioxidants helps to alleviate the peroxidation damage caused by heat stress and facilitates the recovery of heat-tolerant varieties [19]. Storage at high or low temperatures could affect the accumulation of hydroxycinnamic acid derivatives and quercetin and luteolin derivatives, which are strongly related to antioxidant activity and may act as ROS scrubbing agents during bell pepper storage [20]. Previous research has revealed a decline in capsaicin content following heat treatment, which may be due to reduced activities of capsaicinoid synthetase and phenylalanine ammonia-lyase (PAL) [21].

### 2.2. Genes Associated with Tolerance to Temperature Stress in Capsicum

Numerous physiological studies have investigated the response mechanisms of pepper to heat and cold stress. Heat shock proteins (HSPs), calcium and kinase signal transduction, and transcription factor (TF) family members such as Heat shock factors (HSF), WRKY, NAC, CBF, BES, and MADS are observed to be related to temperature stress (Table 1).

HSPs can maintain protein homeostasis and prevent or repair protein misfolding in response to abiotic stress [22]. Plant HSPs also play key roles in protein transport, degradation, and assembly under stress conditions [23]. Heat shock proteins can generally be divided into five families based on their molecular weight: HSP100, 90, 70, 60, and small Heat Shock Proteins (sHSPs). A total of 21 *CaHsp70* genes have been identified in *Capsicum* [24]. The ectopic expression of the cytoplasmic *CaHsp70-2* regulates the expression of heat stress-related genes and improves the thermotolerance of pepper. Knockdown of *CaHSP60-6* confers enhanced sensitivity to heat stress in pepper [25]. The sHSPs of CaHSP16.4 and CaHSP25.9 increase the heat and drought tolerance of pepper by reducing the accumulation of ROS, enhancing the activities of antioxidant enzymes and regulating the expression of stress-related genes [26].

Protein kinases and proteases are important for plant signal transduction, which converts extracellular signals into intracellular responses and mediates various cellular signaling network pathways to regulate related gene expression, plant hormone production, and protein function, and consequently activates plant resistance to stress [27,28]. Calcineurin B-like proteins (CBLs) are specialized calcium sensors that capture calcium signals for transduction to their interacting protein kinases (CIPKs) in response to abiotic stress [29]. The knockout of *CaCIPK13* in pepper enhances the sensitivity of pepper to cold stress and increases MDA content and H_2_O_2_ accumulation [30]. Moreover, CAT, POD, and SOD activities and anthocyanin content decreases. The transcription levels of cold-resistant and anthocyanin-related genes are observed to significantly decrease in *CaCIPK13*-silenced pepper leaves. Furthermore, the interaction between *CaCIPK13* and *CaCBL1/6/7/8* is Ca^2+^-dependent, suggesting that *CaCIPK13* plays an active role in the cold tolerance mechanism through CBL–CIPK signaling.

TFs are key regulators of intracellular and extracellular signal transduction, helping plants adapt to high temperatures and protecting cells from stress damage [31]. HSFs play a key role in plant heat stress and have been identified at the genome-wide level in pepper [32]. *CaHsfA1d* improves heat tolerance by enhancing the expression of genes involved in glutathione synthesis [33], which helps to improve seed heat tolerance and germination vigor [34]. Thus, the expression levels of *CaHSFs* (*CaHSF3*, *CaHSF8*, *CaHSF10*, *CaHSF24*, *CaHSF11*, and *CaHSF18*) in pepper leaves were revealed to significantly increase after 6 h heat treatment [35]. Under high-temperature stress, the expression of *CaPHD37* becomes upregulated in pepper leaves and roots, which may play an important role in antioxidant repair [36], as WRKY could positively respond to high-temperature tolerance in plants. Overexpression of *CaWRKY40* has been shown to reduce the sensitivity of tobacco to high-temperature treatment, while its deletion reduces such tolerance [37]. Under high temperatures and humidity, *CaWRKY40* upregulates the expression of *CaMLO6*, which increases thermotolerance by enhancing the expression of *HSP18* [38]. A total of 104 *CaNAC* genes were identified in pepper, among which the expressions of *CaNAC13*, *CaNAC20*, *CaNAC29*, and *CaNAC53* were significantly upregulated under high-temperature treatment [39]. The resistance to *Ralstonia solanacearum* and heat tolerance of pepper are positively regulated by CaNAC2c via its inducible production and differential interaction with CaHSP70 and CaNAC29 [40]. CaNAC1 positively regulates the expression of *CaPLDα4*, the gene encoding phospholipase D (PLD), resulting in the degradation of membrane phospholipids, increased membrane permeability, and the destruction of cell membrane structure, leading to cold injury in peppers [41]. The ICE1–CBF–COR transcriptional network is the most widely studied pathway in plant response to low temperature [42]. The bHLH transcription factor ICE functions in cold stress response by binding to the promoters of *CBF* genes and some *COR* genes [43]. CBFs can directly bind to C-repeat (CRT/DRE) cis-elements in the promoters of the *cold-responsive* (*COR*) genes and activate their expression, thereby enhancing freeze tolerance in plants [44]. When pepper 5-aminolevulinic acid dehydratase (ALAD) gene *CaALAD* was transformed into *Arabidopsis thaliana*, expression levels of *AtCBF1*, *AtCBF2*, *AtICE2*, *AtCOR15a*, and *AtCOR15b* were promoted, resulting in an enhanced antioxidant system and chlorophyll synthesis and consequently improving cold tolerance under low-temperature stress [45]. The interaction of transcription activator BES1 (BRI1-EMS-SUPPRESSOR 1) with HSPs regulates heat tolerance in pepper [46]. A MADS-box transcription factor CaMADS was shown to play an important role in the response to cold, salt, and osmotic stress. The *CaMADS*-silenced pepper showed impaired plant growth, increased electrolyte leakage (EL) and MDA content, and reduced chlorophyll [47].

**Table 1:** Temperature stress response gene of pepper.

Gene/Gene Family	Description	Functions/Role	Influence by Abiotic Factors	References
*CaPHD37*	Plant homeodomain transcription factor genes	It plays an important role in antioxidant repair	Heat stress	[36]
*CaHSPs*	heat-shock protein	Reduces the accumulation of reactive oxygen species and enhances the activity of antioxidant enzymes	Heat and drought stress	[24,26]
*CaCIPK13*	protein kinase	Regulates cold genes and anthocyanin-related genes	Cold stress	[25,30]
*CaHSFs*	Heat-stimulated transcription factors	The expression of *CaHSF3, CaHSF8, CaHSF10, CaHSF24, CaHSF11, and CaHSF18* was upregulated under heat stress	Heat stress	[24,34,35]
*CaWRKY40*	Contain WRKY domain	Reduces sensitivity to high-temperature treatment, improves heat resistance	Heat stress	[37]
*CaMLO6*	Proteins that target the plasma membrane and could potentially target the ER	*CaMLO6* improves heat resistance by enhancing the expression of *HSP18*, which is associated with heat resistance	Heat stress	[38]
*CaPLDα4*	Gene encoding for phospholipase D	Encodes phospholipase D and accelerates membrane lipid degradation	Cold stress	[41]
*CaNAC*	NAM, ATAF, and CUC transcription factors	The expressions of *CaNAC13*, *CaNAC20*, *CaNAC29,* and *CaNAC53* were upregulated under high-temperature treatment	Heat stress	[39,40]
*CBF*	Transcriptional regulators	*CBF1*, *CBF2,* and *CBF3* were induced to express under low temperature stress	Cold stress	[43,48]
*CaALAD*	Encodes the enzymes essential for chlorophyll biosynthesis	By increasing the expression levels of cold tolerance genes *AtCBF1*, *AtCBF2*, *AtICE2*, *AtCOR15a,* and *AtCOR15b* under low temperature stress, enhancing the antioxidant system, and enhancing chlorophyll synthesis, we could improve plant cold tolerance	Cold stress	[45]
*CaMADS*	Transcriptional regulators	The expression was induced under low-temperature stress	Cold stress	[47]

## 3. Salt Stress

Salt stress affects the absorption, transport, and utilization of mineral ions in roots and the development of roots, stems, leaves, flowers, fruits, and inhibits the photosynthetic process, seed germination, and seedling growth in plants [49].

### 3.1. Physiological Responses to Salt Stress in Capsicum

Pepper is salt-sensitive, especially in the seed germination and seedling growth stages [50]. Increase in salt ion concentration can prolong the germination time of pepper seeds, significantly reduce the germination rate, delay fruit ripening, decrease the number of fruits, and reduce the fruit size, weight, and contents of nutrients such as vitamins B6, B12, and C [51,52]

At present, most studies employ physiological indicators to identify salt tolerance among pepper varieties or materials from different regions. The growth of different pepper genotypes under salt stress exhibits obvious variations. Under low salt concentrations (50 mM), the root dry weight, stem dry weight, leaf number, stem length, stem diameter, total leaf area, chlorophyll, and leaf relative water content of the ‘Goliath’ were reduced, while the growth of the salt-tolerant variety ‘Granada’ was only nearly affected, exhibiting salt tolerance [53].

Pepper has an antioxidant enzyme system to cope with ROS toxicity in salt stress, which enhances antioxidant enzyme (SOD, POD, CAT, and APX) activities similar to those in other plants [54]. The effects of salt stress on the primary and secondary metabolism in pepper depends on different genotypes, fruit parts, and salinity levels [55]. Under salt stress, most phenolics were found in the pericarp, followed by the placenta and seeds, while the sucrose and organic acid contents changed differentially according to different genotypes. Alsharafa [56] found that pepper root growth was significantly inhibited under the treatment of 150 mM NaCl with increased free proline, while long-term salt stress (9 days) enhanced the contents of carotenoids, anthocyanins, H_2_O_2_, MDA, total phenols, and flavonoids. The overexpression of the *PAL1*, *pAMT*, and *PUN1* genes under salt stress promoted the capsaicinoid and dihydrocapsaicinoid production in the roots and leaves of the pepper, but not in the fruits [57].

### 3.2. Genes Associated with Tolerance to Salt Stress in Capsicum

Current research on salt-response genes in plants mainly focuses on genes related to ion balance, antioxidants, and signal transduction, of which ion balance regulator genes are of great value to the salt tolerance of vegetable crops. Some regulatory genes for ion balance have been identified in peppers (Table 2). A total of 42 *CaNHX* genes have been identified in the capsicum genome. Under salt stress, the expressions of *CaNHX1*, *CaNHX3*, *CaNHX4*, *CaNHX5*, and *CaNHX9* are upregulated in roots and remain consistent over time [58]. *CabHLH035* can directly bind to the promoters of *CaSOS1* (*Salt Overly Sensitive 1*) and *CaP5CS* (*pyrroline-5-carboxylate acid synthetase*) and activate their expression, resulting in a downregulated intracellular Na^+^ to K^+^ ratio and higher proline content, and consequently conferring salt tolerance to the pepper [59]. *CaHAK3* and *CaHAK7* are upregulated in pepper roots under low K^+^ or high-salt conditions, suggesting their roles in high-affinity K^+^ uptake transport and maintaining Na^+^/K^+^ balance under salt stress in pepper plants [60].

Antioxidant genes have an important function in vegetable salt tolerance adaptation. When the squamosa-promoter binding protein gene *CaSBP12* was silenced, the accumulation of ROS in pepper leaves was significantly decreased, as were the contents of Na^+^, MDA, and electrical conductivity, suggesting its negative regulation of salt stress tolerance via ROS signaling in the plant [61]. Similarly, the *cysteine protease 15* gene *CaCP15* negatively regulates the tolerance to salt and osmotic stress by reducing antioxidant enzyme activity and negatively regulating stress-related genes under salt and osmotic stress in pepper [62].

The pepper nuclear factor Y subunit B (NFYB) family plays integral roles in vital plant physiological responses, especially under environmental stress. A total of 19 *NFYB* genes have been identified in pepper leaves and roots, with the genes *CaNFYB01*, *CaNFYB18*, and *CaNFYB19* determined to be significantly upregulated at the initial stage of salt stress [63]. The 12-oxo-phytodienoic acid reductase (OPR) family is involved in plant growth, development, and stress responses [64]. Seven OPR genes of *CaOPR1-7* were identified in the pepper genome, of which the expression of *CaOPR6* was significantly induced by several stresses, including low temperature, salt, and pathogen infection [65].

TFs coordinate biochemical and physiological processes that are essential for salt tolerance [66]. Lin et al. [67] found that salt stress, osmotic stress, and ABA treatment induced *CaWRKY27* in pepper, while *CaWRKY27*-transformed arabidopsis and tobacco showed enhanced tolerance to salinity and osmotic stress. ARRs-B TFs are important in regulating cytokinin signaling, abiotic stress resistance, and plant development. Under salt stress, the expressions of *CaARR2* and *CaARR9* are upregulated in the roots of pepper seedlings, while *CaARR3*, *CaARR4*, *CaARR7*, and *CaARR8* expressions decrease in the leaves [68]. The overexpression of *CabZIP25* can enhance the salt tolerance of pepper plants [69].

**Table 2:** Pepper salt stress response gene.

Gene/Gene Family	Description	Functions/Role	Influence by Abiotic Factors	References
*CaNHX*	The Na^+^/H^+^ exchangers	*CaNHX1*, *CaNHX3*, *CaNHX4*, *CaNHX5,* and *CaNHX9* were upregulated in roots under salt stress	Salt stress	[58]
*CabHLH035*	Basic helix–loop–helix (bHLH) proteins	Activation of *CaSOS1* and *CaP5CS* expression and affected intracellular Na^+^ and K^+^ ratio and proline biosynthesis	Salt stress	[59]
*CaHAK*	Potassium transporter gene	*CaHAK3* and *CaHAK7* are upregulated in roots and are candidate genes for high-affinity K^+^ uptake transporters, which may play a key role in maintaining Na^+^/K^+^ balance during pepper salt stress	Salt stress	[60]
*CaSBP12*	SBP-box genes	*CaSBP12* negatively regulates the salt stress tolerance of pepper, which may be related to ROS signal	Salt stress	[61]
*CaCP15*	Cysteine protease	ROS clearance negatively regulates pepper resistance to salt and osmotic stress	Salt stress	[62]
*CaNFYB*	Nuclear factor Y subunit B	The initial stage of salt stress significantly upregulated and plays a central role in initiating the stress defense mechanism	Salt stress	[63]
*CaWRKY27*	Transcription factors, Contain WRKY domain	Regulates defense-related genes to enhance pepper resistance	Salt stress	[67]
*CaARRs-B*	Transcription factors	Under salt stress, *CaARR2* and *CaARR9* were upregulated in roots, while *CaARR3, CaARR4, CaARR7,* and *CaARR8* were downregulated in leaves	Salt stress	[68]
*CabZIP25*	Basic leucine zipper (bZIP) protein	*CabZIP25* improves the plant’s salt tolerance	Salt stress	[69]
*CaOPR6*	12-oxophytoeienoic acid reductase	*CaOPR6* is highly similar to *AtOPR3* in Arabidopsis thaliana, and the expression of *CaOPR6* could be significantly induced by various stresses such as low temperature, salt, and pathogen infection	Cold, salt, pathogenic bacteria	[65]

## 4. Water Stress

Water is the key factor to determining plant physiological processes such as photosynthesis and nutrient transport [70]. Once water stress occurs, it will interfere with various physiological or biochemical processes of plants and exert a serious impact on plant growth.

### 4.1. Physiological Responses to Water Stress in Capsicum

The shallow root system of pepper is sensitive to water supply, with poor nutrient absorption and regeneration capacities [71]. Drought stress can lead to the damage of cell membrane, the degradation of proteins, lipids, and chlorophyll in pepper leaves, and the decrease in intercellular CO_2_ concentration (Ci), stomatal conductance (Gs), and the photosynthetic rate [72], while it causes a significant increase in the contents of capsaicin, soluble carbohydrates, ascorbic acids, catalase (CAT), ascorbate peroxidase (APX), glutathione peroxidase (GPX), polyphenols, and MDA [73]. The flowering stage of pepper is highly sensitive to drought stress. In particular, drought stress can delay the flowering time of pepper, decrease the survival rate of flowers and both the number and size of fruits, and reduce the dry and fresh weight of single fruits as well as the fruit type index [74]. The content of capsaicin is reported to decrease significantly when the fruit is exposed to drought stress, maybe attributed to the possible drought-induced reduction in capsaicin synthase activity [75].

Under flooded conditions, plant root aerobic respiration is inhibited, resulting in a decrease in water absorption, reduced transpiration, failure to keep turgor pressure, and eventually, wilting [76]. In addition, waterlogging can cause curling, wrinkling, and yellowing of leaves, and lead to reduced chlorophyll content, inhibited gas exchange between leaves and the outside environment, and reduced Gs, Tr, and the net photosynthetic rate (Pn) in pepper [77], which induces a significant decline in the plant height, leaf area, and root biomass [78]. The activities of SOD, GPX, CAT, POD, and alcohol dehydrogenase (ADH) in waterlogging-tolerant pepper increase significantly after 5 days of water treatment [79].

### 4.2. Genes Associated with Tolerance to Water Stress in Capsicum

The Abscisic acid (ABA) signaling pathway plays a key role in plant adaptation to water stress. The MAPK cascade is involved in ABA signaling processes, including the regulation of the stomatal opening, seed germination, and antioxidant defenses [80]. Jeong et al. [81] isolated *CaAIMK1* (capsicum ABA-induced mitogen-activated protein (MAP) kinase 1) from ABA-treated pepper leaves, which positively regulates drought stress response through an ABA-dependent pathway (Table 3). *CaAIMK1*-silenced pepper plants exhibited a drought-sensitive phenotype with altered ABA signaling and increased stomatal diameter, while *CaAIMK1*-overexpressed plants exhibited a drought-tolerant phenotype with reduced levels of evaporative water loss and restored sensitivity to ABA and drought. Dehydrins (DHNs) are part of group II of the late-embryogenesis-abundant (LEA) proteins and play an important role in responses to drought, cold, salt, and high-temperature stresses in plants. Under drought stress, *CaDHN3*-overexpressed arabidopsis presented a decrease in MDA content, root length, germination rate, and chlorophyll content but an increase in antioxidant enzyme activities and drought resistance compared with wild-type plants [82]. As such, *CaDHN3* can be induced by ABA and salicylic acid (SA) treatments, which may enhance resistance to drought and salt stresses through the ABA and SA signaling pathways [83]. *CaFAF1* (*Fantastic Four 1*) can be induced by the drought, and its silenced pepper plants exhibited stronger drought tolerance, accompanied by a higher expression of stress-related genes and ABA-mediated stomatal closure. In contrast, *CaFAF1*-silenced pepper plants were sensitive to salt stress, with high electrolyte leakage and lipid peroxidation [84].

Various TFs regulate stress-related genes through ABA-dependent or ABA-independent pathways under water stress. The WRKY, MYB, and bHLH families are involved in regulating the expression of stress-related genes [85]. 172 MYB TFs were identified from pepper, among which *CaDIM1* acted as a positive regulator for the response to the drought stress via the ABA-mediated pathway [86]. *CabHLH18* was cloned from a highly waterlogging-tolerant pepper variety ‘ZHC2’, and its overexpression lines had greater root length, plant height, and fresh weight, as well as the higher amino acid, Pro, soluble sugar, root activity, and SOD activity than that of wild-type plants, while MDA content was lower under waterlogging conditions [87]. *CaNAC46* responded to various stresses and improved pepper resistance to drought and salt stresses by regulating the antioxidant enzyme content [88].

Ubiquitin is a key regulatory factor in numerous cellular functions (e.g., protein classification and hormone signaling) via protein degradation. Cho et al. [89] isolated the pepper *peptide U-box protein 1* (*CaPUB1*) from water-stressed peppers, which could be induced under different abiotic stress conditions such as drought, salt, and cold stress. *CaPUB1-*overexpressed arabidopsis plants exhibited drought sensitivity and acted as a negative regulator for response to the drought stress.

**Table 3:** Water stress response gene of pepper.

Gene/Gene Family	Description	Functions/Role	Influence by Abiotic Factors	References
*CaAIMK1*	Aba-induced MAP kinase 1	ABA-dependent pathways	Drought stress	[81]
*CaDHN3*	DHN gene of Y1SK2 type	The resistance to drought stress and salt stress enhanced by ABA and SA signaling pathways	Drought and salt stress	[83]
*CaFAF1*	Capsicum annuum Fantastic Four 1	Drought tolerance of pepper was negatively adjusted, and salt tolerance was positively adjusted	Drought and salt stress	[84]
*CaDIM1*	Capsicum annuum Drought Induced MYB 1	*CaDIM1* regulates ABA-mediated gene expression	Drought stress	[86]
*CabHLH18*	bHLH transcription factor	Amino acids, proline, soluble sugar, root activity, and SOD activity were increased	Flooding stress	[87]
*CaNAC46*	NAC transcription factor	Regulates the content of antioxidant enzymes and increases the resistance of plants to drought and salt stress	Drought and salt stress	[88]
*CaPUB1*	Pepper U-box E3 Ubiquitin Ligase	Negatively regulates drought stress response	Drought and salt stress	[89]

## 5. Heavy Metal Stress

Heavy metal pollution generally refers to the pollution caused by metals and their compounds, with a density of more than 4.5 g/cm^3^ in the environment. The residues of heavy metals such as Mn, Cu, Ni, Co, Cd, Fe, Zn, and Hg in soil can be absorbed by plants, leading to growth and development retardation and reduced yield and quality [90]. At present, research on plant tolerance to heavy metal stress generally focuses on seed germination, root development, photosynthesis, nutrient absorption, and oxidation stress.

### 5.1. Physiological Responses to Heavy Metal Stress in Capsicum

With the increase in heavy metal concentration (Cu concentration ≥ 100 mg/kg or Cd concentration ≥ 5 mg/kg), the final germination percentage (FGP), mean germination time (MGT), germination index (GI), coefficient of velocity of germination (CVG), germination rate index (GRI), and germination rate (GR) of bell pepper seeds gradually decrease [91]. Cu stress was reported to inhibit plant seed germination mainly by changing the external osmotic pressure of seeds and consequently impairing their water uptake capacity [92]. At higher concentrations of vanadium (≥30 mg/L), the growth of pepper seedlings was inhibited, with decreased leaf gas exchange parameters and pigment content and increased soluble sugar and Pro contents and antioxidant enzyme activities [93]. Excessive Ni was reported to induce leaf chlorosis, root deformity, photosynthesis inhibition, and biomass reduction in pepper seedlings. Additionally, Ni accumulation in pepper leaves and roots reduced the contents of nitrogen, potassium, and phosphorus [94]. Todirascu-Ciornea and Dumitru [95] found that under (CH_3_COO)_2_Pb, FeSO_4_, and SnCl_2_ stress, the activities of SOD, CAT, and POX enzymes in pepper seedlings significantly increased. Hasan et al. [96] found that Cd(NO_3_)_2_ and Pb(NO_3_)_2_ treatment induced a decrease in the chlorophyll and carotenoid contents and a significant decrease in the photosynthetic rate and Tr in pepper.

### 5.2. Genes Associated with Tolerance to Heavy Metal Stress in Capsicum

Under Cd stress, the expression of the pepper *phytochelatin synthase* (*PCS*) gene increased significantly, and it had higher expression level in root than that in stem and fruit. Furthermore, its expression level was higher in pepper genotypes with higher Cd-accumulation ability (Table 4) [97]. The heavy metal transporter ATPase (HMA or P1B-ATPase) gene *CaHMA1* was a determinant for accumulation of Cd content in pepper fruits, and the knockdown of *CaHMA1* caused reduced Cd content in fruits [98]. Tao et al. [99] isolated an early photoinducible protein gene *CaELIP* from a pepper cDNA library that was induced by heavy metal stress, which could be induced by ROS produced via excessive Cu stress. Under heavy metal stress, the biosynthesis of metabolites in the phenylpropane pathway was regulated by key enzymes such as PAL, cinnamic acid 4-hydroxylase (C4H), 4-coumaric acid CoA ligase (4CL), and Chalcone synthase (CHS) [100]. The upregulated expression of *PAL*, *C4H*, and *4CL* genes could promote the biosynthesis of lignin, flavonoids, and phenolic acids, which was conducive to enhancing plant tolerance to heavy metal stress by removing ROS and slowing down membrane peroxidation [101]. Under high zinc stress (Zn(NO_3_)_2_ ≥ 10 mM), the *PAL* expression in bell pepper leaves increased significantly [102]. Under the stress of Cd^2+^, Cu^2+^, Pb^2+^, Zn^2+^, and Fe^3+^, the expression levels of some *CaMYB* genes were reported to be upregulated, with the most significant upregulation of *pairing homologous 1* (*PH-1*), *PH-13*, and *PH-15* in pepper roots, indicating the sensitivity of MYB family members to heavy metals [103].

**Table 4:** Heavy metal response genes of capsicum.

Gene/Gene Family	Description	Functions/Role	Influence by Abiotic Factors	References
*PCS*	phytochelatin	The expression of the *PCS* gene was significantly increased under Cd stress	Metal Cd stress	[97]
*CaHMA1*	Transporter	Binding to metal ions	Metal Cd stress	[98]
*CaELIP*	Early light inducible protein gene	ROS could induce the expression of CaELIP	Metal Cu stress	[99]
*PAL*	Phenylalanine ammonia lyase gene	The expression level increased under stress	Metal Zn stress	[102]
*CaMYB*	transcription factor	The expression of *PH-1*, *PH-13,* and *PH-15* genes was upregulated	Cd^2+^, Cu^2+^, Pb^2+^, Zn^2+^ and Fe^3+^ stress	[103]

## 6. Crosstalk Between Abiotic and Biotic Stress Responses

The signaling pathways involved in plant resistance to biotic and abiotic stresses share some common modules, including those involved in TFs, ROS, signaling pathways (calcium, ABA, jasmonate, and MAP cascade signaling) [104,105,106]. These signaling pathways help plants quickly adapt to changing environments under biotic and abiotic stresses.

The gene expressions induced by various stresses mainly occur at the level of transcriptional regulation. The activation or inhibition of stress-related gene expression by TFs is an important process of plant stress responses [107]. ERF, MYB, NAC, and WRKY have been found to play important roles in biotic and abiotic stresses in plants [108,109]. MYC and other transcription factor family genes are involved in the resistance response to abiotic stress (e.g., drought, salt, and low-temperature stress) and biological stress (e.g., pathogen infection). In pepper, the expressions of NAC TFs (*CaNAC72*, *CaNAC92*, and *CaNAC102*) are upregulated under drought, salt, and high-temperature stress [110]. Oh et al. [111] rapidly induced *CaNAC1* after infection with bacterial or viral pathogens, including *Xanthomonas axonopodis* pv. *Glycines, Pseudomonas syringae* pv. *syringae 61,* and *Pepper mild mottling virus* (PMMV). Zheng et al. [112] identified 62 WRKY genes in the latest pepper genome. *CaWRKY13*, *CaWRKY15*, *CaWRKY34*, and *CaWRKY42* all showed differential expressions under high temperature and cold stress. *CaWRKY10, CaWRKY13*, and *CaWRKY15* were upregulated at all stages of *Cucumber mosaic virus* (CMV) inoculation, and *CaWRKY27* positively regulated the resistance to bacterial wilt (BW). The expression of *CaWRKY30* was upregulated after infection with various pathogens, including avirulent *Meloidogyne incognita*, *Tobacco mosaic virus* (TMV), *Ralstonia solanacerum*, and *Phytophthora capsici* Leonian.

The *Mildew Locus O* (*MLO*) gene is transcriptionally induced by both biotic and abiotic stress. Pepper *MLO* functions as a general negative regulator of plant immunity against hemi-biotrophic bacterial pathogens, such as *Xanthomonas campestris* pv. *Vesicatoria*, *Pseudomonas syringae* pv. *tomato* and *Hyaloperonospora arabidopsidis*. Additionally, in *Capsicum*, *CaMLO2* was strongly induced in pepper leaves exposed to ABA and drought and acted as a negative regulator of ABA signaling that suppressed water loss from leaves under drought conditions [113]. Thus, the MAP kinase (MAPK/MPK) cascade plays a crucial role in regulating defense responses against biotic and abiotic stresses [114]. The MEKK1/MKK2/MPK4/MPK6 cascade is involved in signaling under biotic and abiotic stress conditions and also has a key function in crosstalk signaling between abiotic and biotic stress mechanisms [115]. Capsicum *CaMAPK1* plays an important role in the regulation of resistance to bacterial wilt infection and heat tolerance and is regulated by SA, JA, and ABA signals [116]. ROS also has a critical function in crosstalk signaling in abiotic and biological stresses. Adaptive mechanisms in plants are initiated by the combined action of ROS and stress-induced signaling pathways, which together activate and respond immediately to a variety of external stimuli. Ali et al. [117] reported that *CaChiVI2-*silenced peppers exhibited increased H_2_O_2_ and O^2−^ concentrations after high temperature and anthracnose treatment and were more vulnerable to injury compared to the control.

## 7. Stress Relief Measures

Over the past few years, researchers have systematically studied the indicators of various abiotic stresses in pepper at the physiological and molecular levels and also explored how to alleviate those stresses. Among them, seed soaking and foliar spraying are commonly used measures.

### 7.1. Relief Measures to Temperature Stress

Plant growth regulators (PGRs) alleviate abiotic stress and alter plant growth and development by inducing changes in cellular and physiological processes [118]. Preet et al. [119] found that plant height, leaf area, photosynthetic efficiency, membrane thermostability, pollen vitality, and fruit set percent under heat stress were significantly higher than those of the control by the foliar spraying of SA and 2,4-epibrassinolide (EBR) solution. Methyl jasmonate (MeJA) can be used to reduce the leaf damage caused by high temperatures [120] and protect seeds from browning under cold stress. Similarly, 5-Aminolevulinic acid (ALA) treatment can improve the germination rate and total germination rate of pepper seeds in low-temperature environments and increase seedling uniformity [121]. Under low-temperature stress, spermidine plays a stabilizing role in the membrane by increasing antioxidant enzyme activities and reducing the permeability of electrolytes, which alleviates the cold damage and reduces the cold sensitivity of pepper [122]. In addition, CaCl_2_ treatment was found to effectively regulate the activities of antioxidant enzymes, reduce the accumulation of O^2−^ and H_2_O_2_ and the expression and activity of *PLC* and *DGK* genes, inhibit the degradation of membrane lipid components, improve relative conductivity, and maintain the integrity of the cell membrane, thereby inhibiting the occurrence of cold injury in pepper [123]. Spraying 200 uM melatonin at the seedling stage is shown to improve cellular structures of pepper roots under low temperatures, increase the content of osmotic regulatory substances (soluble sugar and soluble protein) and the activities of antioxidant enzymes (SOD, POD, CAT, APX, DHAR, and MDHAR), and enhance the tolerance of pepper seedlings to low temperatures [124]. Foliar spraying of SA could also increase the germination rate of pepper seeds under high-temperature stress, improve photosynthetic capacity, and alleviate the damage to pepper seedlings caused by high temperatures [16]. In addition, selecting appropriate rootstocks for grafting could also alleviate the damage caused by high-temperature stress. Lamuyo-type sweet pepper ‘Herminio F1’ (VA) grafted onto an American *Capsicum annuum* L. genotype ‘A25’ showed significantly lower EL and increased plant height, leaf area, and the number and yield of commercial fruits per plant under heat stress compared with ungrafted varieties (VA) [125].

### 7.2. Relief Measures to Salt Stress

Numerous studies have applied exogenous hormones (gibberellic acid, MeJA, EBR, and SA), osmoregulatory substances (Pro, glycine betaine, and trehalose), antioxidants (ascorbic acid, glutathione, and tocopherol), signaling molecules (NO and H_2_O_2_), and trace elements (Se and Si) to reduce salt stress damage [126,127]. These exogenous protective agents not only improve the saline–alkali resistance of pepper but promote the growth and development of pepper. The use of SA was reported to ameliorate the negative effects on the growth of pepper seedlings at 50 mM and 100 mM NaCl [128]. Exogenous sodium selenite, for example, can promote pepper seed germination, alleviate the inhibition of salt stress on seedling growth, promote root and above-ground growth, and significantly reduce the contents of O^2−^ and MDA to reduce the effects of salt stress [129]. Foliar spraying with a certain concentration of H_2_O_2_ can improve the growth and water-use efficiency of bell peppers and reduce the damage by salt stress [130]. Under high salt stress, seaweed extract can increase the accumulation of potassium and phosphorus, slow down the accumulation of sodium, and improve the ion balance, yield, and post-harvest quality of pepper [131]. In addition, biological control measures could be adopted to alleviate the plant damage caused by salt stress. Arbuscular mycorrhizal fungi combined with seaweed extracts [132] and biochar [133] could promote pepper seedling growth and alleviate salt-stress damage (Selvakumar and Thamizhiniyan 2011). Additionally, Bacillus subtilis WU-9 can alleviate salt-stress damage to seedlings [134], while grafting can significantly increase plant height, the fresh weight of stems and roots, photosynthetic rate, Gs, water use efficiency, and pepper yield [135].

### 7.3. Relief Measures to Drought and Flood Stress

The physiological changes adopted by plants to adapt to drought stress include the regulation of osmotic and hormone substances and the enhancement of the antioxidant system [136]. The addition of SA, NO, ALA, and MeJA can each improve the drought resistance of pepper. Under drought stress, the foliar spraying of SA is reported to decrease leaf conductivity but increase leaf area, leaf relative water content, fruit longitudinal and transverse diameters, and antioxidant capacity, alleviating the negative effects of drought stress on pepper [137]. Spraying exogenous MeJA on pepper leaves can increase antioxidant enzymes and alcohol dehydrogenase activities and Pro and soluble sugar contents, decrease relative conductivity, MDA and hydroxyl radical accumulation, lactate dehydrogenase activity, lactic acid, and acetaldehyde accumulation, and maintain high malate dehydrogenase and succinate dehydrogenase activities and aerobic respiratory metabolism. Spraying before waterlogging is recommended to alleviate the damage of waterlogging on pepper [138]. Brassinosteroids (BRs) alleviate the harmful effects of water stress on pepper by enhancing the antioxidant system [139]. The exogenous application of γ-aminobutyric acid (GABA) can improve the stem length, root length, fresh/dry weight, root ratio, chlorophyll content, soluble protein content, soluble sugar content, total Pro content, and antioxidant enzyme (CAT, APX, POD, and SOD) content of pepper [140].

### 7.4. Relief Measures to Heavy Metal Stress

Current management strategies for soil-heavy metal pollution include engineering, phytoremediation, and agronomic control methods. Kaya et al. [141] found that application of silicon (Si) could reduce Cd content in leaves, improve the antioxidant defense system and photosynthetic activity, and increase the contents of hydrogen sulfide (H_2_S), nitric oxide (NO), and Pro, finally resulting in enhanced tolerance to Cd in the pepper. NO scavenger could completely abolish the mitigation effect of Si, while H_2_S scavenger partially removed the mitigation effect of Si to Cd poisoning. Under heavy metal stress, melatonin treatment significantly increased root length, root surface area, and root volume, and promoted the growth of pepper seedlings. Melatonin increased leaf photosynthetic capacity and the activities of antioxidant enzymes (SOD, GR, APX, CAT, POD, GST, GPX, DHAR, and MDHAR), reduced the accumulation of H_2_O_2_, O^2−^, and MDA, and inhibited oxidative damage, effectively enhancing the tolerance of pepper seedlings to vanadium, chromium (Cr), Cd, and nickel (Ni) [142]. Plant growth-promoting rhizobacteria (PGPR) can alleviate the effects of cadmium (30 ppm), Plumbum (Pb) (150 ppm), and the combination of Cd and Pb stresses on pepper, and promote the growth and development of pepper in heavy metal-contaminated soil [143].

## 8. QTL Mapping for Abiotic Stresses in the *Capsicum* Genome

Several QTLs have been reported in pepper for biotic stresses such as *Pepper yellow leaf curl virus* (PepYLCV) [144], *Phytophthora capsici* [145], and bacterial wilt [146]. QTL mapping analysis of pepper agronomic traits generally focuses on fruit [147,148], pungency degree [149], flowering time [150], and pollen fertility [151].

Numerous QTLs for abiotic stress tolerance—including low temperature, drought, and salt stress—have been identified in crops such as wheat, tomato, and rape [152,153,154]. However, there is a lack of research on the QTL mapping of abiotic stress for pepper. This may be because the effects of abiotic stresses in pepper involve multiple physiological and biochemical reactions and gene expression regulatory networks, with interactions between different abiotic stresses. Furthermore, phenotypic changes under abiotic stress are complex and difficult to accurately identify and quantify, making QTLs difficult to locate and analyze.

## 9. Molecular Breeding for Resistance and Tolerance Against Stresses in *Capsicum*

### 9.1. Molecular Marker-Assisted Breeding

Molecular marker-assisted breeding can improve the accuracy and efficiency of breeding and ensure the genetic stability and consistency of varieties while avoiding the effects of environmental factors. The development of molecular markers, the construction of genetic linkage maps, and the identification of QTLs and genes are prerequisites for molecular breeding. Multiple molecular genetic maps have been constructed using isolated populations of cultivated pepper and different wild pepper species [155]. Paran et al. [156] constructed a genetic map of pepper by integrating existing data containing 2262 markers covering 1832 cM. Molecular markers have been used to analyze the genetic diversity of pepper germplasm resources [157], screen materials resistant to *Phytophthora capsici* [158], identify resistance to *Potato virus Y* (PVY) [159], and identify QTLs for important traits [160,161]. They are also employed in fine mapping, candidate gene identification, and marker-assisted breeding for pepper leaf color, ripeness, fruit shape, color, ventricle number, spiciness, nuclear sterility, cytoplasmic male sterility recovery, anti-anthracnose, and viral diseases in pepper [162]. However, there is a lack of similar applications for abiotic stress to pepper.

### 9.2. Gene Editing Breeding

The development of de novo domestication of wild-related species of crops using gene editing technology can recapture lost but desirable traits, including nutritional traits, traits related to stress resistance and yield potential, and other agriculturally valuable traits [163]. The genome sequencing of pepper was completed in 2014, laying the foundation for research on gene editing technology [164]. Li et al. [165] first identified CRISPR/Cas9 editing sites 5′-NGG-3′ and 5′-NAG-3′ at a genome-wide level in pepper ‘Zunla-1’ and evaluated their specificity through genome-wide alignment. The authors determined that this set of highly specific sites has broad applicability and helps to minimize the off-target effects of CRISPR/Cas9 in pepper. Kim et al. [166] used protoplasts from pepper leaves and calli as receptors for ribonucleoprotein (RNP) transfection and achieved the editing of *CaMLO2—*a target gene with broad spectrum resistance to powdery mildew pathogens—in pepper cultivars ‘CM334’ and ‘Dempsey’. Park et al. [167] employed pBAtC as the CRISPR/Cas9 gene editing vector to optimize the concentration of the glufosinate-ammonium (PPT) screening agent. The results identified Agrobacterium tumefaciens strain GV3101 as the optimal strain to transform pepper cultivars ‘CM334’ and ‘Dempsey’. CRISPR/Cas9 technology has been used to edit the phytoene desaturase gene to reduce the essential photosynthetic pigments (chlorophyll a, chlorophyll b, and carotenoids) in pepper [168].

At present, gene editing has not been employed in pepper resistance breeding to abiotic stress. There is a lack of efficient genetic transformation technology suitable for multiple pepper genotypes. In addition, nonlinear genetic effects such as phenotypic interactions and pleiotropy pose challenges for the direct application of gene editing to continuously improve and create new varieties by manipulating key genes with superior agronomic value through gene editing [169].

## 10. Limitations and Prospects

Although pepper varieties in resistance to abiotic stress have been identified, it is far from meeting the commercial demand for resistant cultivars. The research process in this area is slower than that on high yield, high quality, and resistance to diseases and pests. Due to the complexity and variability of environmental factors, the effects of abiotic stresses on pepper growth and development require further in-depth study. There is a lack of large-scale productivity evaluations in the field and systematic identification and screening for excellent pepper germplasm resources resistant to abiotic stress. Molecular markers and relevant QTLs associated with abiotic stress are still limited in pepper. Although molecular breeding technology has developed rapidly, theoretical and methodological research on its combination with conventional breeding technology should be strengthened. With the rapid development of modern biotechnology, the changes in levels of transcription, translation, metabolism, and phenotype in response to abiotic stress could be comprehensively analyzed to understand the molecular regulatory network of pepper response to abiotic stress. This review provides a reference to further excavate the key genes in response to abiotic stresses and provides genetic resources for the application of molecular breeding technology in *Capsicum*.
